# Gossypin-Loaded Ethosome Gel for Cutaneous Administration: A Preliminary Study on Melanoma Cells

**DOI:** 10.3390/antiox14020186

**Published:** 2025-02-05

**Authors:** Agnese Bondi, Walter Pula, Mascia Benedusi, Giulia Trinchera, Anna Baldisserotto, Stefano Manfredini, Maria Grazia Ortore, Alessia Pepe, Paolo Mariani, Marc C. A. Stuart, Giuseppe Valacchi, Elisabetta Esposito

**Affiliations:** 1 Department of Chemical, Pharmaceutical and Agricultural Sciences, University of Ferrara, I-44121 Ferrara, Italy; agnese.bondi@unife.it (A.B.); walter.pula@unife.it (W.P.); 2 Department of Neurosciences and Rehabilitation, University of Ferrara, I-44121 Ferrara, Italy; 3 Department of Environmental Sciences and Prevention, University of Ferrara, I-44121 Ferrara, Italy; giulia.trinchera@unife.it; 4 Department of Life Science and Biotechnology, University of Ferrara, I-44121 Ferrara, Italy; anna.baldisserotto@unife.it (A.B.); stefano.manfredini@unife.it (S.M.); 5 Department of Life and Environmental Sciences, Marche Polytechnic University, I-60131 Ancona, Italy; m.g.ortore@staff.univpm.it (M.G.O.); a.pepe@staff.univpm.it (A.P.); p.mariani@staff.univpm.it (P.M.); 6 Facility Manager Electron Microscopy, University of Groningen, 9747 AG Groningen, The Netherlands; m.c.a.stuart@rug.nl; 7 Animal Science Department, Plants for Human Health Institute, NC State University, NC Research Campus, Kannapolis, NC 28081, USA; 8 Department of Food and Nutrition, Kyung Hee University, Seoul 02447, Republic of Korea

**Keywords:** gossypin, skin, ethosome, xanthan gum, A735 melanoma cell lines

## Abstract

A preformulative study was conducted to produce and characterize ethosomes for the transdermal delivery of gossypin. This plant-derived compound possesses many pharmacological properties, including antitumoral potential. Ethosome dispersions were designed as transdermal delivery systems for gossypin, employing two different production procedures. The evaluation of vesicle size distribution by photon correlation spectroscopy, morphology by cryogenic transmission electron microscopy, and gossypin entrapment capacity, as well as in vitro release and permeation by vertical diffusion cells, enabled us to select a production strategy based on the injection of a phosphatidylcholine ethanolic solution in water. Indeed, vesicles prepared by this method were almost unilamellar and measured roughly 150 nm mean diameter while displaying an entrapment capacity higher than 94%. Moreover, vesicles prepared by the ethanol injection method enabled us to control gossypin release and to improve its permeation with respect to the solution of the drug. To obtain semi-solid forms suitable for cutaneous gossypin administration, ethosome dispersions were thickened with 0.5% *w*/*w* xanthan gum, selected by a spreadability test. These ethosome gels were then further characterized by small- and wide-angle X-ray scattering, while their antioxidant activity was demonstrated in vitro by a radical scavenging assay. Finally, in vitro biological studies were conducted on A375 melanoma cell lines. Namely, wound healing and cell migration assays confirmed the potential antitumoral effect of gossypin, especially when loaded in the selected ethosomal gel. The promising results suggest further investigation of the potential of gossypin-loaded ethosomal gel in the treatment of melanoma.

## 1. Introduction

Cutaneous melanoma is the most aggressive form of skin cancer; indeed, based on epidemiological data, in 2020 more than 300,000 new melanoma cases and about 60,000 deaths occurred [1]. Moreover, a 50% increase in new cases and deaths over the next two decades has also been estimated. The incidence of this human cancer varies greatly between countries, not only due to differences in skin phenotype and sun exposure, but also due to easier or lesser access to early diagnosis and treatments [2,3]. Notwithstanding recent significant advancements in the clinical management of advanced melanoma through targeted therapies and immunotherapy, treatment failures, severe adverse effects, and resistance to current therapies persist [4,5]. These challenges emphasize the need for alternative treatment options. One possible solution can be found in drugs of natural origin. Indeed, nature represents a very rich resource of bioactives, particularly derived from plants, with promising therapeutic properties as anticancer drugs, or as adjuvants [6,7]. Nevertheless, due to their low stability, scarce solubility and bioavailability, their use in cancer treatment is still limited. In this regard, the development of nanotechnological delivery systems could represent a smart approach to overcome these drawbacks. Gossypin (GOS) is a flavanol glucoside naturally found in the roots and flowers of various hibiscus plants, such as *Hibiscus esculentus*, *Hibiscus vitifolius*, and *Gossypium indicum*, all belonging to the Malvaceae family. GOS has garnered significant scientific interest due to its diverse therapeutic potential. Research has explored its various beneficial effects, including anti-diabetic properties, anti-inflammatory, antiviral, and anti-allergic effects, cardiovascular system benefits, central nervous system effects, and anti-proliferative/antitumor as well as antioxidant activity [8,9]. GOS exhibits antitumor activity by interfering with cellular processes. Specifically, it can arrest the cell cycle and inhibit proliferation, these effects are achieved through the modulation of various cellular pathways. One key mechanism involves the inhibition of NF-κB, a protein complex that plays a crucial role in inflammation and cell survival. By inhibiting NF-κB, GOS can reduce inflammation and promote tumor cell death. In addition, GOS inhibits TNF-induced MMP9 expression: Tumor necrosis factor (TNF) can stimulate the production of matrix metalloproteinase 9 (MMP9), an enzyme that degrades the extracellular matrix and facilitates tumor invasion and metastasis. By inhibiting TNF-induced MMP9 expression, GOS can hinder tumor spread [9]. In this context, GOS has been proposed for the treatment of melanoma [10,11]. Rajkumar and colleagues have pioneered research into the potential transdermal application of GOS using vesicular gel formulations, specifically pro-niosomal gels [11]. The transdermal administration of compounds enables the following: (i) sustained drug release, (ii) improved patient compliance, (iii) reduced side effects, (iv) bypassing first-pass metabolism, and (v) improved patient comfort [11,12]. Thus, in the present study, the possibility of achieving transdermal delivery of GOS and its possible application for cutaneous melanoma was investigated by loading it in ethosomes (ETs). ETs, designed by Touitou and colleagues in 2000 [13], are biocompatible nanovesicular systems composed essentially of phosphatidylcholine (PC), water, and ethanol in a relatively high percentage (20–45%). The presence of ethanol enhances stability and flexibility and increases the ability to solubilize lipophilic active ingredients as compared to classical liposomes [14,15,16]. Furthermore, ethanol is a key element in enabling the vesicles to penetrate through the skin layers. Due to its well-known penetration-enhancing behavior, ethanol can transiently disorganize the stratum corneum barrier, allowing transdermal penetration by nanovesicles, made more malleable by ethanol itself [14,17]. In a recent study by our research group [18], TEM images confirmed the ability of ETs to reach the deeper skin layers intact when applied on an ex vivo model consisting of human skin explants. For this reason, ETs emerge as an effective delivery system for the treatment of diseases involving deep skin layers, such as melanoma.

This investigation aimed to develop and characterize ETs for the topical delivery of GOS to treat cutaneous melanoma. Two distinct methodologies were employed to produce ETs, which were subsequently compared to identify the optimal method for GOS loading. The resulting colloidal dispersions were comprehensively characterized, evaluating vesicle size distribution and morphology and GOS encapsulation efficiency, as well as in vitro release and permeation. To enhance the applicability and residence time of the formulations on the skin, the ET dispersions were thickened using xanthan gum (x-gum). The obtained ET gels were then further characterized by small-angle X-ray diffraction and a spreadability test and for their antioxidant activity. Finally, the biological activity of the optimized formulations was assessed through in vitro studies on A375 melanoma cell lines.

## 2. Materials and Methods

### 2.1. Materials

Gossypin (GOS, (2-(3,4-dihydroxyphenyl)-3,5,7-trihydroxy-8-[(2S,3R,4S,5S,6R)-3,4,5-trihydroxy-6-(hydroxymethyl)oxan-2-yl]oxychromen-4-one) was obtained from Cayman Chemical Company (Ann Arbor, MI, USA). Phosphatidylcholine (PC) (purity 94%) from soybean was procured from A.C.E.F. Spa. Xanthan gum (x-gum) was obtained from Merck Life Science S.r.l. (Milan, Italy). Nylon membranes and STRAT-M^®^ membranes were acquired from Merck Life Science S.r.l. (Milan, Italy). Solvents were HPLC-grade, and all other chemicals were analytical-grade.

### 2.2. Preparation of Ethosomes

In the present investigation, two different ET preparation methods were employed, namely the water injection (WI) and the ethanol injection (EI) methods.

#### 2.2.1. Water Injection (WI)

The WI method consists of the gradual addition of water drop-by-drop, continuously, to a PC solution in ethanol (30 mg/mL) kept under magnetic stirring (IKA RCT basic, IKA^®^-Werke^®^ GmbH and Co. KG, Staufen, Germany) at 750 rpm. The final water/ethanol ratio is 70:30 (*v*/*v*). Once the addition is complete, stirring is maintained for 30 min. The PC solution is prepared beforehand, solubilizing the lipid in ethanol at room temperature through magnetic stirring. The entire procedure takes place at room temperature. In case of GOS-loaded ETs, the drug was previously solubilized in the PC–ethanol solution, while to avoid photodegradation all steps were performed in the dark [18].

#### 2.2.2. Ethanol Injection (EI)

The EI method differs from the WI method by reversing the mixing mode of the two phases. To be specific, the PC–ethanol solution (30 mg/mL), is gradually added to water kept under magnetic stirring at 750 rpm. The final water/ethanol ratio is always 70:30 (*v*/*v*) and, once the addition is completed, magnetic stirring is maintained for 30 min. All steps take place at room temperature. As previously described, the GOS-loaded ETs could be prepared by previous drug solubilization in the PC–ethanol solution, protected from the light.

### 2.3. Photon Correlation Spectroscopy (PCS) and Zeta Potential Measurements

ET size distribution was evaluated using a Zetasizer Nano-S90 (Malvern Instr., Malvern, UK). The instrument is equipped with a 5 mW helium–neon laser that produces a wavelength output of 633 nm. The analyses were conducted at 25 °C at a 90° angle and with a 120 s equilibration time. The samples were diluted 1:10 (*v*/*v*) with bi-distilled water. The size distribution was obtained by the “CONTIN” method [19]. Each analysis was carried out in triplicate within 60 days postproduction of the ET samples. Zeta potential values were acquired by measuring electrophoretic mobility according to the Hemholtz–Smoluchowski equation [20]. The results were expressed as means ± standard deviation (s.d.).

### 2.4. Morphological Characterization by Cryogenic Transmission Electron Microscopy (Cryo-TEM)

Three microliters of sample were placed on a holy carbon-coated grid (Quantifoil 3.5/1), blotted and vitrified in ethane (Vitrobot). Frozen hydrated grids were observed using a Tecnai T20 electron microscope (FEI Co., Eindhoven, The Netherlands) operating at 200 keV. Images were recorded with a slow-scan CCD camera under low-dose conditions [21,22].

### 2.5. Entrapment Capacity Evaluation

To quantify the entrapment of the active agent within ET, the ultrafiltration method was employed. Specifically, one day after preparation, 500 µL of ET formulations were put in a Microcon centrifugal filter unit with a YM-10 membrane (NMWCO 10 kDa, Sigma-Aldrich, St. Louis, MO, USA) and then subjected to ultrafiltration (Spectrafuge™ 24D Digital Microcentrifuge, Infitek Inc., Spokane, WA, USA) at a centrifugal force of 4000 rpm for 20 min. The retentate vesicular fraction was disrupted by dilution in ethanol (1:10 *v*/*v*) and magnetic stirring for 30 min. An aliquot (100 µL) of the total dispersion was treated likewise to determine the actual drug content within the formulation. Before the HPLC analysis, the disaggregated samples were filtered using nylon syringe filters (0.22 µm). Finally, the entrapment capacity (EC), was calculated as:

EC = G/T_G_ × 100 (1)

In the equation, G represents the amount of GOS retained by the vesicles and T_G_ refers to the real GOS content in the whole formulation.

### 2.6. Franz Cells Experiments

To perform the in vitro release test (IVRT) and in vitro permeability test (IVPT), Franz vertical cells were employed (orifice diameter 0.9 cm, PermeGear Inc., Hellertown, PA, USA), assembled with appropriate membranes according to the intended study. Specifically, NY membranes were used for the IVRT while STRAT-M^®^ membrane was employed for the IVPT. The NY membranes’ function was only to divide the donor and the receptor compartments as an inert support, while the STRAT-M^®^ membrane had the function of simulating the stratum corneum barrier properties. The receptor compartment was filled with 5 mL of ethanol/water 50:50 (*v*/*v*) to assure sink conditions. The receiving phase was kept under magnetic stirring at 500 rpm and 32 °C throughout the duration of the experiment. Before Franz cell assembly, the membranes were rehydrated by immersion in ethanol/water 50:50 *v*/*v* for 1 h. The donor compartment was filled with 1 mL of the test formulation and then sealed to avoid evaporation. The experiment was carried out by periodic withdrawals (1 per hour between 0 and 24 h) of 0.5 mL from the receiving compartment which were then analyzed using HPLC to quantify the amount of drug that passed through the membrane. Each removed sample was replaced with an equal volume of fresh receiving phase.

#### 2.6.1. IVRT

For data analysis, in the case of the IVRT, the amount of released drug (expressed as µg/cm^2^) was graphed as a function of time [23]. From the linear portion of the release profile, “R” and “A” values were obtained. In specific, “R”, the release rate, was obtained from the slope of the curve, while “A” was the amount of drug released at the final sampling point [24]. Furthermore, to elucidate the mechanism of GOS release, data were analyzed according to mathematical models such as the zero-order kinetics (cumulative % drug released vs. time), first-order kinetics (log cumulative % drug remaining vs. time), Higuchi (cumulative % drug released vs. square root of time), and Peppas (log cumulative % drug released vs. log time) models. The suitability of the model fits was verified using the DDsolver add-in for Excel 2016 (Version 2312 Build 16.0.17126.20126).

#### 2.6.2. IVPT

In the case of the IVPT, for data analysis, the amount of permeated drug (expressed as µg/cm^2^) was plotted as a function of time [25]. According to Fick’s law, under sink conditions, the drug concentration in the receptor compartment is negligible compared with that in the donor compartment, which is equal to the concentration gradient. The linear portion of the curve described the steady-state permeation through the skin, from which we could calculate a set of descriptive parameters for the process. The steady-state flux of the drug per unit area “Jss” (µg cm^−2^ h^−1^) is the slope of the curve and can be described by the following equation:

Jss = P × Cd × D/e(2)

“P” is the partition coefficient, “Cd” is the drug concentration in the donor compartment, “D” is the drug diffusion coefficient, and “e” is the thickness of the membrane [26]. Drug permeability coefficient “Kp” values were calculated from the Jss values according to the following equation:

Kp = Jss/Cd(3)

Lag time, “T_lag_”, (time needed to reach steady-state conditions) could be obtained from the intersection of the line with the *x*-axis, and the “D” value was calculated from T_lag_ by the following equation:

T_lag_ = e^2^/6 × D (4)

At last, P was achieved considering the following equation:

Kp = D × P/e(5)

### 2.7. HPLC Analysis

The HPLC analyses were carried out using Perkin Elmer Series 200 HPLC Systems (PerkinElmer, Waltham, MA, USA) equipped with a micropump, an autosampler, and an UV detector operating at a wavelength of 260 nm. A stainless-steel C-18 reverse-phase column (15 × 0.46 cm) packed with 5 µm particles (Hypersil BDSC18 Thermo Fisher Scientific S.p.A., Milan, Italy) was eluted at a flow rate of 1 mL/min with a mobile phase composed of acetonitrile/water 30:70 *v*/*v* added with 0.1% TFA. Injection volume was 5 µL and retention time was 3 min.

### 2.8. Preparation and Characterization of ET Gel

ET dispersions were thickened using x-gum. Specifically, one day after the preparation of the ET (loaded or unloaded with drugs), ET gels were prepared by the direct addition of x-gum to the colloidal dispersion kept under magnetic stirring. To avoid lump formation, the addition was performed gradually under stirring until the system turned uniform. Different concentrations of x-gum were studied (i.e., 0.5, 0.75, and 1% *w*/*w*) for ET prepared both with the EI and WI methods. The resulting gels were tested for spreadability.

#### Spreadability Test

The test was based on evaluating the area occupied by a formulation when a constant weight was applied to it [27]. Precisely, 100 mg of gel was placed in the center of a glass Petri dish (3 cm diameter) and then subjected to pressure by a glass dish carrying a 50 g mass. Thereafter, we waited for 10 s before measuring the diameter of the surface occupied by the formulation. Spreadability value (S) was measured with the following equation:

S = m × l/t,(6)

where “m” is the applied weight (expressed in g), “t” is the application time (10 s), and “l” is the diameter (expressed in cm) of the area occupied by the gel. The trial was performed three times for each tested formulation.

### 2.9. X-Ray Diffraction

Small-angle X-ray scattering (SAXS) experiments were performed using the SAXS beamline of an Elettra Synchrotron (Trieste, Italy) [28] and were part of the CERIC proposal, number 20242026. The samples were located in an apposite quartz capillary with a diameter of 1.5 mm, thermostatted within a KPR (Peltier heating/cooling) sample holder (Anton Paar, Graz, Austria). Results at ambient temperature (25 °C) and at skin temperature (32 °C) were reported. In addition, two samples (ET_WI_ and ET-GOS_WI_) were measured in more detail in a wider temperature range (from 15 to 60 °C, every 5 °C). For each sample, the exposition time was 10 sec and for each condition 18 repeating frames were considered. Two-dimensional (2D) data were corrected for background, detector efficiency, and sample transmission and then radially averaged to derive the differential scattering cross-section—more simply, scattered intensity I(Q)—as a function of the modulus of the scattering vector, Q, defined as 4π sinθ/*λ* (where 2θ is the scattering angle and *λ* is the X-ray wavelength). The explored Q-range was between 0.1 and 5 nm^−^^1^. The detector used for the measurement was a 2D Pilatus3 1M Detector System (Dectris, Baden-Daettwil, Switzerland) with a pixel size of 172 *×* 172 μm^2^ and a discrete energy of 8 keV (corresponding to the beam wavelength of 0.154 nm). An additional wide-angle X-ray scattering (WAXS) detector was used to simultaneously monitor diffraction patterns in the high-angle range from 8 to 17 nm^−^^1^.

### 2.10. Antioxidant Activity

#### 1,1-Diphenyl-2-Picrylhydrazil (DPPH) Radical Scavenging Assay

By using DPPH assays, the ability of an antioxidant substance to convert the stable free radical DPPH to 1,1-diphenyl-2-picrylhydrazyl by hydrogen donation was obtained. The reaction was monitored and measured by a UV–Vis spectrophotometer (UV/VIS ONDA Touch UV-31 Scan Spectrophotometer, Sinergica Soluzioni S.r.l., Milan, Italy), evaluating the reduction in absorbance at 517 nm, corresponding to a colorimetric change from violet to light yellow, as previously described [29]. Solutions at different concentrations were prepared for each sample (SOL GOS, ET-GOS_EI_, ET-GOS_EI_-0.5% gel) and 0.75 mL of each of these solutions was added to 1.5 mL of DPPH in methanolic solution. From the calibration curve obtained for each sample, the IC_50_ (µg/mL) value was obtained, given as the mean of three independent experiments with standard deviation.

### 2.11. Biological Activity

#### 2.11.1. 3-(4,5-Dimethylthiazol-2-yl)-2,5-Diphenyltetrazolium Bromide (MTT) Assay

A375 melanoma cells were cultured in Dulbecco’s modified Eagle’s medium high glucose (DMEM), (Lonza, Milan, Italy), supplemented with 10% FBS (fetal bovine serum), 100 U/mL penicillin, 100 μg/mL streptomycin, and 2 mM L-glutamine. Cells were incubated at 37 °C for 24 h in 95% air/5% CO_2_ until 80% confluence was reached.

For cell treatments, the different vehicles were at first dissolved in cell culture medium to obtain the stock solutions containing GOS 300 µg/mL, then further diluted to reach different concentrations of GOS (from 0.5 to 50 μg/mL).

Specifically, A375 cells were grown in 96-well plates at a density of 2 × 10^4^ cells/well in 200 μL of media for the MTT assays. The day after, the cells were treated with different doses of unloaded and GOS-loaded formulations with GOS concentrations ranging from 0.5 μg/mL to 50 μg/mL for 24 and 48 h. Then, the treatment was completely removed and 50 μL of serum-free media and 50 μL MTT (0.5 mg/mL) were added. After 3 h of incubation (37 °C, 5% CO_2_), the formed insoluble purple formazan crystals were dissolved in 100 μL of dimethyl sulfoxide (DMSO) for 15 min at 37 °C, 5% CO_2_. After 5 min of shaking, the solution absorbance was read with a spectrophotometer at 590 nm, using 670 nm as the reference wavelength. Finally, the values were converted into a percentage of cell viability, compared to CTRL (T0) [30].

#### 2.11.2. Wound Healing Assay

A375 melanoma cells were grown on 24-well plates at a density of 5 × 10^4^ cells/well in complete DMEM medium to a confluent monolayer. The day after, the cells were first mechanically scratched with a 200 μL sterile pipette tip and then cellular debris were removed by two washes with 1× PBS. Based on the MTT assay results, A375 cells were immediately exposed to 0.5 μg/mL of GOS-loaded and unloaded formulations, followed by incubation at 37 °C. Finally, images of the scratches were recorded at different time points (viz. 0, 3, 6, 12, 24 and 48 h post- scratch) and the wound healing rate was analyzed by means of ImageJ software 1.54f (National Institutes of Health, Bethesda, MD, USA) and compared to the wound area at T0 [31].

#### 2.11.3. Cell Migration Assay

2.2 × 10^6^ A375 melanoma cells were seeded in 10 cm^2^ Petri dishes and pretreated with 0.5 μg/mL of GOS-loaded and unloaded formulations for 24 h; then, 100,000 cells were resuspended in 400 μL of serum-free media and seeded in 8 μm pore-size transwells (Falcon^®^ Permeable Support for 12-well Plate with 8.0 µm Transparent PET Membrane, Sterile; Falcon, Becton Dickson, Oxnard, CA, USA) coated with 0.15 mg/mL bovine collagen IV. After 30 min, 1100 μL of complete media was added to the bottom of each well, acting as a chemoattractant. After 24 h, the transwell inserts were first fixed with 70% ethanol (for 10 min), then stained with 0.02% of Coomassie Blue (15 min) and rinsed with double-distilled water. The remaining A375 cells that did not migrate from the upper part of the transwell were gently removed with a cotton swab, while pictures of 5 randomly selected fields were acquired (at 20× magnification). The number of migrated cells present in the five fields/well was counted using the ImageJ program. Data were reported as percentage of migrated cells compared to CTRL condition [32].

## 3. Results and Discussion

### 3.1. Preparation of Ethosomes

ETs were chosen as the transdermal delivery system for GOS. ETs are, in fact, biocompatible vesicular systems able to reach the deepest layers of the skin due to the presence of ethanol, a known penetration enhancer, which is able to act synergistically with PC, promoting passage through the skin barrier [18].

Furthermore, the high ethanol percentage in their composition facilitates the solubilization of lipophilic drugs. Notably, in the present investigation, two different ET preparation methods were compared. The WI method has previously been employed by our research group [32,33,34], demonstrating the ability to produce stable vesicles with an average diameter of approximately 200 nm and a homogeneous size distribution. In this method, bi-distilled water is gradually added to a PC–ethanol solution under continuous stirring. Conversely, in the case of the EI method, the PC–ethanol solution is gradually added to water under continuous stirring. Table 1 presents the composition of the ET dispersions prepared by water injection (ET_WI_) or ethanol injection (ET_EI_).

Both methods resulted in homogeneous dispersions with a milky and stable appearance over time. Noteworthily, the ET_EI_ was more transparent than ET_WI_.

### 3.2. Size Distribution and Stability

Size characterization was evaluated by PCS one day after ET preparation. The results reported in Table 2 indicate that the preparation method influenced ET size. The vesicle mean diameter of ET_EI_, expressed as Z-Average, was 50–60 nm smaller compared to ET_WI_, while both methods yielded homogeneous size distributions, as evidenced by the dispersity index being below 0.2. To elucidate the size difference, several factors can be considered. It is established that vesicles form spontaneously through the self-assembly of PC units at the ethanol–water interface, a process facilitated by mixing. Specifically, mutual diffusion between the ethanol and water solvents occurs at the interface, compelling the PC units to associate in bilayered structures, which subsequently fold to form vesicles. It can be postulated that in the case of the EI method, wherein the organic solution is gradually added to an excess of water, rapid depletion of the organic solvent occurs, resulting in a reduction in the diffusion length. As previously observed in microfluidic systems [35], rapid depletion of the organic solvent leads to an acceleration of bilayer closure, thus producing smaller vesicles. Conversely, with the WI method, where water is added to an excess of PC–ethanol solution, the length of the diffusion layer tends to increase, and vesiculation occurs more gradually, favoring larger structures.

The size stability of the formulations was also assessed by repeating the analysis by PCS after 60 days of storage at 22 °C and protected from light. As shown in Table 2, a slight increase in the average diameter over time was detected, while the size distribution remained homogenous, with a dispersity index increase after 2 months in the case of ET_WI_. In the Supplementary Materials (Figure S1), the time course of the Z-Average and PdI values is graphically shown.

### 3.3. Morphological Characterization

**Figure 1:**
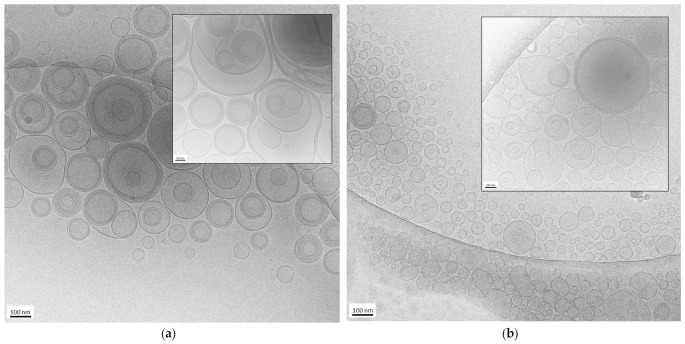
Cryo-transmission electron microscopy (cryo-TEM) images of ET_WI_ (**a**) and ET_EI_ (**b**). Bar corresponds to 100 nm in panel (**a**,**b**) and 50 nm in inset of panel (**a**,**b**).

The morphology of the nanovesicles produced by the EI and WI methods was assessed by cryo-TEM. Some images of ET_EI_ and ET_WI_ are shown in Figure 1.

In both cases, the formation of mostly spherical or ovoidal vesicles was observed, in agreement with PCS analysis data. In the case of ET_WI_ (panel a and inset), the prevalence of multilamellar and oligolamellar vesicles is observable. In contrast, in the case of ET_EI_ (panel b and inset), a majority of unilamellar and oligolamellar vesicles is evident, suggesting the different preparation methods influenced vesicle morphology. It is possible to assume that the rapid depletion of the organic solvent in the ethanol injection method leads to quicker vesiculation, thereby hindering the formation of multilamellar structures. On the contrary, the addition of water to the excess PC–ethanol solution not only promotes more gradual vesiculation, leading to the formation of larger structures, but also the assembly of multiple bilayers, resulting in a predominance of oligolamellar and multilamellar vesicles.

The mean diameter of the vesicles shown in the cryo-TEM images is 170 ± 60 nm in the case of ET_WI_ (Figure 1a) and 88 ± 20 nm in the case of ET_EI_ (Figure 1b), confirming that the EI method enables us to obtain smaller vesicles compared to the WI method, as observed by PCS. Nevertheless, the vesicle size appears smaller compared to the PCS values reported in Table 1. In this regard, several factors should be considered. On the one hand, PCS determines vesicle size by analyzing the diffusion of scattered light. On the other hand, since cryo-TEM provides localized information about samples, limiting the view, the measurements of size obtained using this technique can be unreliable compared to the results for the same parameter obtained by PCS. Indeed, distances and geometries may appear distorted due to the limitations of the imaging process, leading to over- or underestimations. Despite these limitations, cryo-TEM remains valuable for visualizing nanoparticles in near-native conditions, enabling morphological characterization [36].

### 3.4. Preparation of GOS-Loaded Ethosomes

To prepare GOS-loaded ETs, both the EI and WI methods were investigated under previous GOS solubilization in a PC–ethanol solution. Since the limit of GOS solubility in the PC–ethanol solution was 1 mg/mL, ET-GOS allowed us to load 0.3 mg/mL of drug (Table 1). The GOS presence conferred to the dispersions a yellowish color, with a more transparent aspect in the case of ET-GOS_EI_ compared to ET-GOS_WI_. In case of the GOS-loaded formulations, the presence of the drug did not affect the size of the nanovesicles, showing the same size reduction between ET-GOS_EI_ and ET-GOS_WI_. The dispersity index was always below 0.2, suggesting a homogeneous size distribution also present in loaded formulations (Table 2).

### 3.5. Entrapment Capacity Evaluation

EC was evaluated through the ultrafiltration method, allowing us to separate the vesicular fraction from the aqueous dispersing phase. Retentate fractions and whole dispersions were disaggregated by dilution in ethanol, while HPLC analysis enabled us to quantify GOS concentration. As reported in Table 2, GOS was almost completely associated with nanovesicles. Moreover, a higher EC was found in the case of ET-GOS_EI_ compared to ET-GOS_WI_, evidencing the higher potential of the EI method for GOS loading.

To gain information about the loading of GOS, the Zeta potential values of the unloaded and loaded ETs were evaluated, and the results are reported in Table S1 in the Supplementary Materials. In agreement with previous findings, the Zeta potential values ranged between −20 and −25 mV, indicating a high ET stability. Indeed, the Zeta potential parameter provides an indicator of colloidal system stability due to the degree of repulsion between the vesicles in the dispersing phase [18]. The negative sign is related to ethanol, conferring a negative charge on the vesicle surface. GOS loading did not affect Zeta potential compared to the unloaded ETs, suggesting its high association within the vesicles, in agreement with the EC values.

### 3.6. IVRT

**Figure 2:**
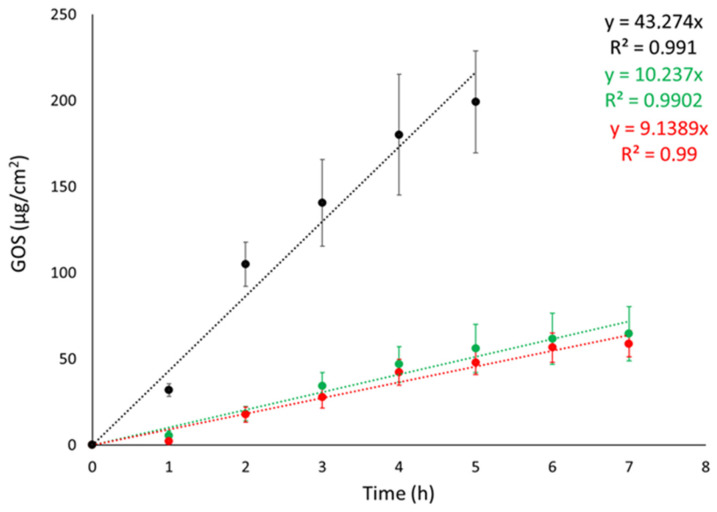
GOS release kinetic data from ET-GOS_EI_ (red circles), ET-GOS_WI_ (green circles), and SOL–GOS (black circles), as determined by Franz cells associated with nylon membrane. Data are means of 6 independent experiments ± s.d.

Franz cell apparatus was employed to conduct IVRTs with the objective of evaluating the ETs’ capability to control GOS release. Synthetic nylon membrane was selected as an inert support to separate the upper and lower compartments. In the study, the release of GOS from ET-GOS_EI_ and ET-GOS_WI_ was compared to a 30:70 ethanol/water GOS solution (SOL-GOS) containing GOS 0.3 mg/mL. As expected, the ETs achieved a better control of GOS release compared to SOL-GOS. Indeed, as shown in Figure 2, GOS release from SOL-GOS was much faster compared to the ET forms. No differences were observed between the release profiles obtained in the case of ET-GOS_WI_ and ET-GOS_EI_.

Zero-order plot, first-order plot, Higuchi plot and Peppas plots were drawn to clarify the mechanism of drug release from the ETs. Table 3 shows the R^2^ values obtained by plotting the release profiles of the formulations according to the different mathematical models [37]. For both ET-GOS_WI_ and ET-GOS_EI_, the profile was better described by the first-order kinetic model, while the n values obtained from the Peppas plot reveal a non-Fickian diffusion mechanism.

### 3.7. IVPT

**Figure 3:**
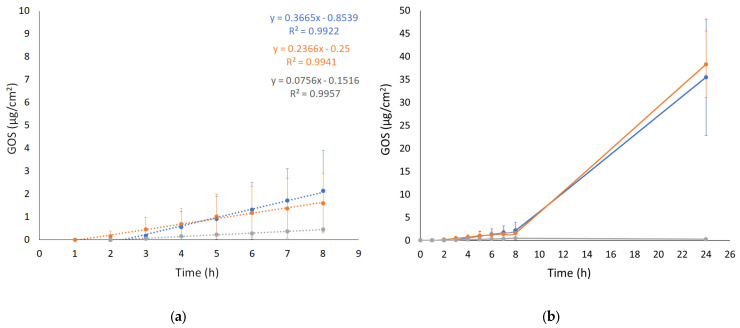
GOS diffusion profiles from ET GOS_EI_ (blue circles), ET GOS_WI_ (orange circles), and SOL GOS (gray circles), as determined by Franz cells associated with STRAT-M^®^ membrane. (**a**) 0–8 h linearized profile and (**b**) 0–24 h diffusion. Data are means of 6 independent experiments ± s.d.

The IVPT was performed using Franz cell apparatus associated with a STRAT-M membrane. STRAT-M is a synthetic membrane whose lipophilicity and porosity can mimic the barrier properties of the stratum corneum. Specifically, STRAT-M is a multilayered polymeric membrane consisting of two polyether sulfone layers overlapping a polyolefin lowermost layer impregnated with synthetic lipids to further provide a skin resemblance [38]. The aim of the test was to evaluate the ability of the ETs to enhance GOS diffusion in vitro, comparing ET-GOS prepared with the EI and WI methods to GOS free in solution. The diffusion profiles (0–24 h) are shown in Figure 3. The passive GOS diffusion across the membrane occurs through two main steps. The first one is described by P, reflecting the preferential distribution of the drug between the membrane/skin or the vehicle. The second step, on the other side, is described by D and Kp, referring to GOS passage across the membrane/skin and depending on the drug and vehicle physicochemical characteristics [39,40]. The calculated diffusion parameters are reported in Table 4.

As shown in Figure 3 (panel a), the diffusion of GOS from SOL-GOS was much slower compared to the ET formulations, confirming the potential of the vesicular systems in increasing the diffusion of the GOS drug through the skin. A certain lag time (Tlag) was found in all cases, following the order ET-GOS_EI_ > SOL-GOS > ET-GOS_WI_. The Kp values of ET-GOS_WI_ and ET-GOS_EI_ were, respectively, 3- and 4.5-fold higher compared to SOL-GOS. Interestingly, the P value obtained in the case of the ET-GOS_EI_ was 3.35-fold higher compared to ET-GOS_WI_, suggesting a greater GOS partitioning towards the membrane. The amount of drug permeated after 24 h (A value in Table 4) in the case of SOL-GOS was only 0.26 μg/cm^2^, roughly 140-fold lower compared to the amount permeated in the cases of ET-GOS_WI_ and ET-GOS_EI_.

### 3.8. Ethosomal Gel Preparation and Characterization

In order to facilitate topical application, the ETs were thickened by x-gum, a natural biocompatible polysaccharide widely used in various fields, including food, pharmaceutics, and cosmetics [41]. ET gels were prepared by the direct addiction of x-gum (0.5, 0.75 or 1% *w*/*w*) to an ethosomal dispersion (both ET_WI_ and ET_EI_) under stirring, until complete gum dispersion was achieved. Homogeneous gels were obtained in all cases, without macroscopical differences between the ET_WI_ gel and ET_EI_ gel, showing an increase in thickening and opacity as a function of the x-gum amount. The gel spreadability values evaluated for the different gels are reported in Table 5. As expected, the percentage of x-gum was inversely related to the spreadability values. No differences between the ET_WI_ gel and ET_EI_ gel were detected, and 0.5% x-gum was selected, leading to the highest gel spreadability.

The spreadability parameter is particularly relevant to ensure the smooth and comfortable application of the product to the skin and easy extrusion from the container.

### 3.9. Small- and Wide-Angle X-Ray Scattering

**Figure 4:**
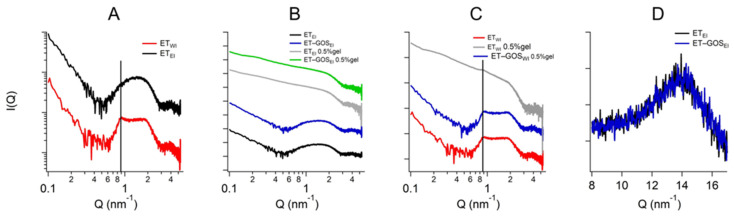
Comparison among the SAXS profiles of ET_EI_ and ET_WI_ (**A**), ET_EI_, ET-GOS_EI_, ET-GOS_EI_ 0.5% gel (**B**), and ET-GOS_WI_ 0.5% gel (**C**). Black vertical line in panel C indicates peak in Q position of 0.87 nm^−^^1^ visible in each profile. Comparison between WAXS profiles of ET_EI_ and ET-GOS_EI_ (**D**).

X-ray scattering allowed us to investigate the structure of the ETs prepared by the EI and WI methods, as well as their semi-solid forms, also considering the effect of temperature. The characteristic SAXS profile of these vesicular nanosystems measured at 25 °C is depicted in Figure 4A, wherein the broad band between 0.6 and 1.3 nm^−^^1^ indicates the presence of the lipid bilayer of the ETs. The primary distinction pertains to a small Bragg peak observed at Q = 0.87 nm^−^^1^ for ET_WI_ (see the black vertical line). This observation likely reflects the multilamellar vesicles characterizing this sample, corroborating the results obtained by cryo-TEM and providing an interlamellar distance of 7.22 nm, which is consistent with previous findings on similar drug-delivery systems [42]. Moreover, the peak remains stable also in the presence of x-gum (ET_WI_ 0.5% gel) and GOS (ET-GOS_WI_ 0.5% gel) (Figure 4C), indicating that the lamellar organization is maintained. Concerning ET_EI_, Figure 4B shows the absence of changes when x-gum and GOS are present in the system. The SAXS data obtained in the presence of x-gum (green and gray curves) illustrate a profile masking the band of the lipid bilayer because of the high signal of the polymer in this component. However, the presence of the lamellar bilayer in ET_WI_ is still visible in Figure 4C, as indicated by the Bragg peak highlighted by the vertical line.

Regarding the WAXS measurements, a few results are reported in Figure 4D. Indeed, the scattering profiles characterizing the different samples are very similar (see the data reported in Figure 4D for ET_EI_ and ET-GOS_EI_ samples), suggesting the absence of structural differences in the single-lipid composition of the vesicle bilayers. Moreover, both the SAXS and WAXS profiles of the ET_EI_ and ET-GOS_EI_ samples are similar to their plurethosome profiles, previously investigated by our research group [42].

**Figure 5:**
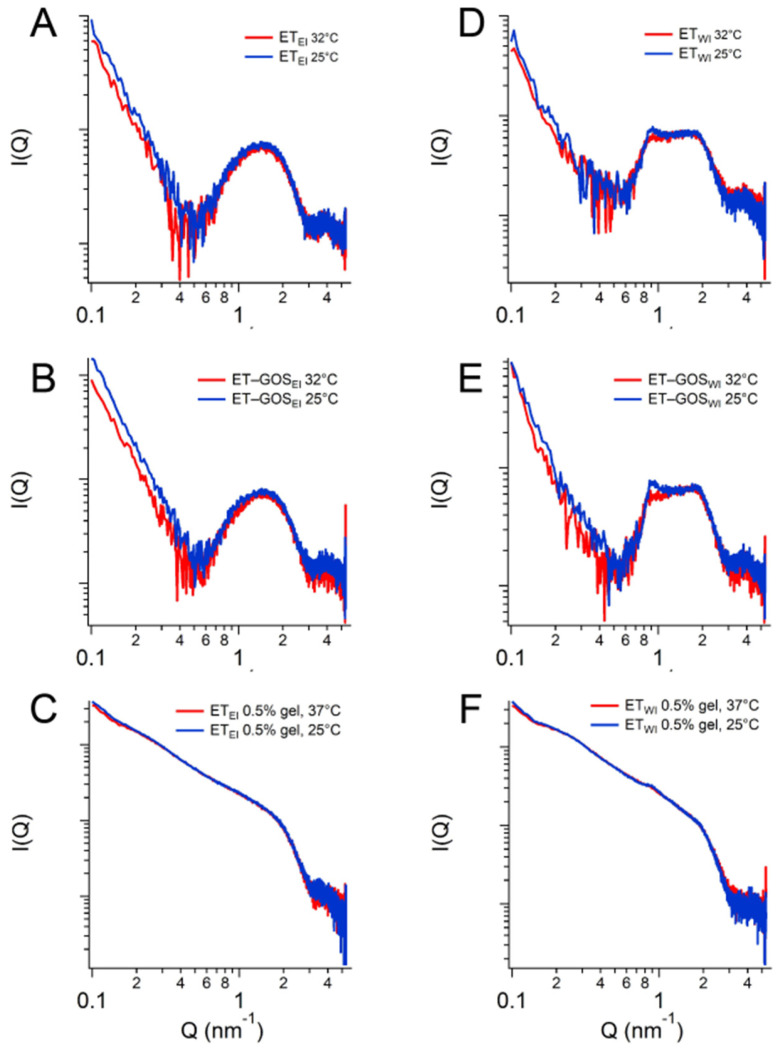
SAXS profiles of ET_EI_ (**A**), ET-GOS_EI_ (**B**), ET_EI_ 0.5% gel (**C**), ET_WI_ (**D**), ET-GOS_WI_ (**E**), and ET_WI_ 0.5% gel (**F**) at 25 °C and 32 °C.

SAXS results obtained as a function of temperature are illustrated in Figure 5. In particular, a comparison between the conditions at 25 °C and 32 °C is presented, demonstrating the stability of the ET formulation both at storage and skin temperature, with superposable profiles. A minor difference is observed for the formulation ET_WI_, where SAXS data reveals a lower intensity of the peak at Q = 0.87 nm^−^^1^. Based on this observation, a temperature scan from 15 to 60 °C was performed for both the ET_WI_ and ET-GOS_WI_ samples to investigate potential alterations in vesicle structure due to the storage of these formulations, as reported in the Supplementary Materials.

Based on the obtained results, the EI method was selected for subsequent tests. ET-GOS_EI_ was in fact characterized by (a) smaller vesicles compared to ET-GOS_WI_, possibly improving penetration through the skin and cell uptake, (b) higher GOS EC values, and (c) a higher GOS permeability coefficient.

### 3.10. Antioxidant Activity

In order to assess the antioxidant activity of GOS loaded in ETs, a DPPH test was performed. The antioxidant activity of free GOS in solution (SOL GOS) was compared with GOS loaded in ET-GOS_EI_ and in the thickened formulation ET-GOS-0.5% gel. The IC_50_ values are shown in Table 6, compared with an ethanolic solution of ascorbic acid as a reference antioxidant (SOL-AA).

By comparing the IC_50_ values, it is evident that the antioxidant activity of GOS is maintained even when loaded within ETs, and is in the same micromolar range as the reference compound. Conversely, the IC_50_ values obtained for ET-GOS_EI_ are significantly lower compared to ET-GOS_EI_-0.5% gel (*p* < 0.005). This result can be attributed to the 3D structure of the gel, slowing the GOS release from the vesicles, possibly resulting in a prolonged effect of the drug. On the other hand, empty formulations (i.e., ET_EI_ and ET_EI_-0.5% gel) tested as controls did not show any antioxidant activity, confirming that the activity detected for the drug-loaded formulations is ascribable to GOS.

### 3.11. IVRTs and IVPTs of GOS-Loaded Ethosome Gel

In order to gain information about the release and permeation of GOS from the semi-solid formulation to be applied on the skin, IVRTs and IVPTs were performed and the results are shown in Figure S3 and Table S2 (Supplementary Materials). ET-GOS_EI_-0.5% gel was able to control GOS release, exhibiting a release rate roughly 2.5-fold slower compared to ET-GOS_EI_ dispersion and 12-fold slower than the GOS solution, as expected due to the thickened structure of the gel. The IVPT demonstrated that in the case of ET-GOS_EI_-0.5% gel, T_lag_ was the longest and D was the lowest, while the Kp value of GOS was the highest compared to the other formulations. Particularly, Kp was almost superposable to ET-GOS_EI_, suggesting the capability of the thickened formulation to maintain the transdermal efficacy of the ETs, promoting GOS permeation through the skin-like membrane. Going forward, to effectively predict in vivo behavior, preclinical studies will be necessary.

### 3.12. Biological Activity Studies

Wound healing and migration assays were conducted on A375 melanoma cells to investigate the potential of GOS loaded in ETs and in ET gels in the treatment of melanoma.

Indeed, some studies indicate the anticancer effects of GOS and its role in melanoma progression [10]. Particularly, ET-GOS_EI_, ET-GOS_EI_-0.5% gel, and SOL-GOS were evaluated and compared with their unloaded forms, ET_EI_, ET-0.5% gel, and EtOH/H_2_O 30:70 *v*/*v* (EtOH/H_2_O).

#### 3.12.1. MTT Assay

First, the cytotoxicity of the formulations was determined by an MTT assay on an A375 melanoma cell line. Specifically, cells were treated with different formulation amounts (i.e., 0.5, 1, 2.5, 5, 10, 20, and 50 µg/mL), evaluating viability after 24 h and 48 h from the treatments.

**Figure 6:**
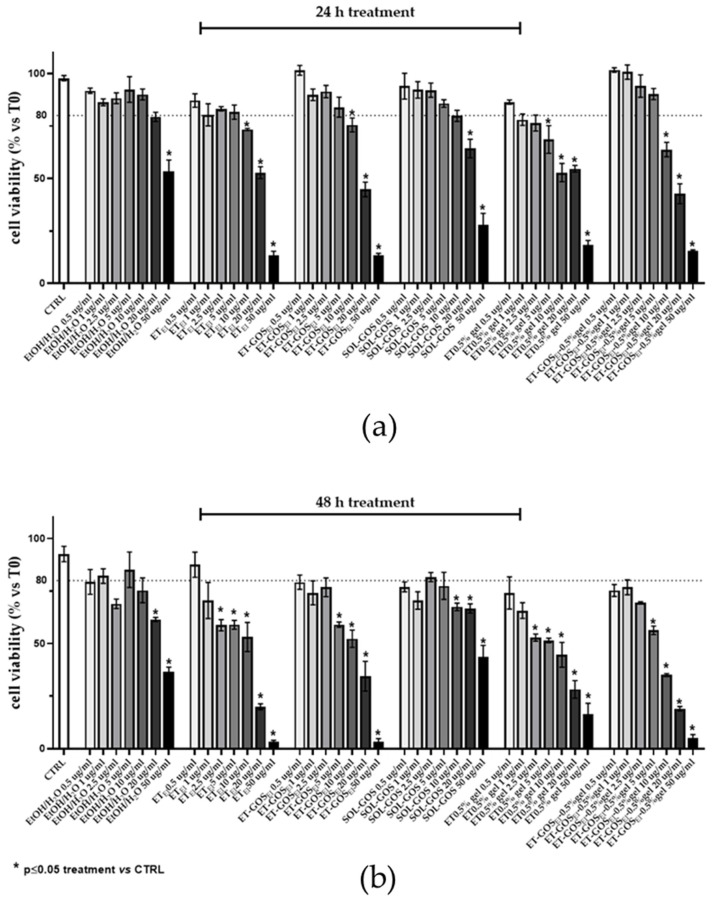
A375 melanoma cell viability evaluated by MTT test after 24 h (**a**) or 48 h (**b**) of treatment with GOS-loaded or unloaded formulations. Data are results of three independent experiments performed in triplicate. Data appear as mean ± SE. * *p* < 0.05 by two-way ANOVA (post hoc test: Tukey test) of Ctrl vs. treated cells.

As depicted in Figure 6, while after 24 h cell viability decreased only for doses higher than 2.5 µg/mL (panel a), after 48 h a decrease (viability under 80% threshold) in cell viability was detected at lower dosages of GOS compared to untreated cells (panel b). Therefore, a 0.5 µg/mL amount of GOS-loaded and unloaded formulations were selected for further experiments, with this being a non-toxic concentration that did not induce a reduction in cell viability higher than 25%. The MTT assay was utilized as a preliminary assessment to select the dose for the subsequent experiments on cell migration, ensuring the administered dose did not induce cellular toxicity.

#### 3.12.2. Wound Healing Assay

**Figure 7:**
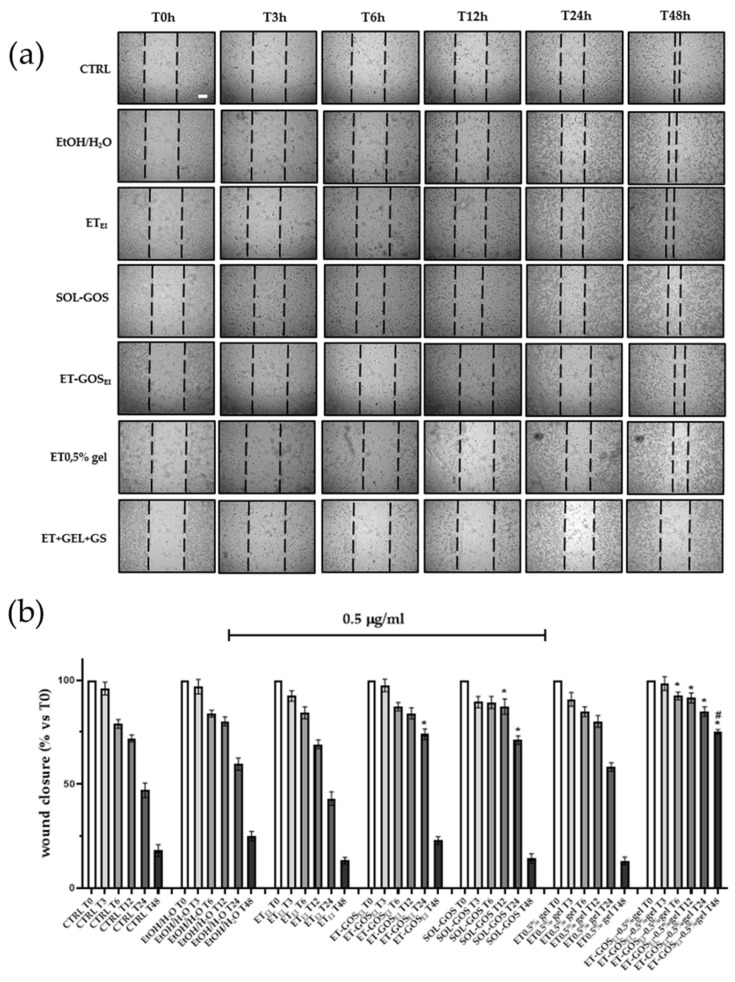
Effect of treatment with 0.5 µg/mL of GOS-loaded or unloaded formulations on wound closure in A375 melanoma cells. (**a**) Scratch was performed on confluent monolayer of A375 cells and different images were taken to measure wound area at different time points (0, 3, 6, 12, 24, and 48 h; scale bar 200 μm). (**b**) Quantification of the wound area at each time point via ImageJ. Data are shown as percent of 0 h. Data are results of 3 independent experiments performed in triplicate. * *p* < 0.05 vs. Ctrl; # *p* < 0.05 ET-GOSEI-0.5% gel vs. treatment with other two formulations containing GOS by one-way ANOVA.

To evaluate the possible effect of GOS-loaded and unloaded formulations on melanoma cell migration, a wound healing assay was performed on an A375 cell line. As depicted in Figure 7, cells treated with SOL-GOS, ET-GOS_EI_, and ET-GOS_EI_-0.5% gel showed delayed wound closure compared to the control cells. In addition, when GOS was loaded in ET-GOS_EI_, the wound closure rate was slightly slower than in SOL-GOS, confirming the controlled-release potential of the nanocarrier. Furthermore, when the cells were treated with ET-GOS_EI_-0.5% gel, wound closure was even more delayed, with 75% still open after 48 h. This result is not only statistically significant compared to the control, but also significantly different from those obtained upon A375 cells’ treatment with SOL-GOS and ET-GOS_EI_. Taken together, these wound healing data firstly confirm the potential antitumoral effect of GOS and also strengthen the hypothesis concerning the ability of the semi-solid formulation to prolong and further control the release of the loaded drug.

#### 3.12.3. Transwell Migration Assay

**Figure 8:**
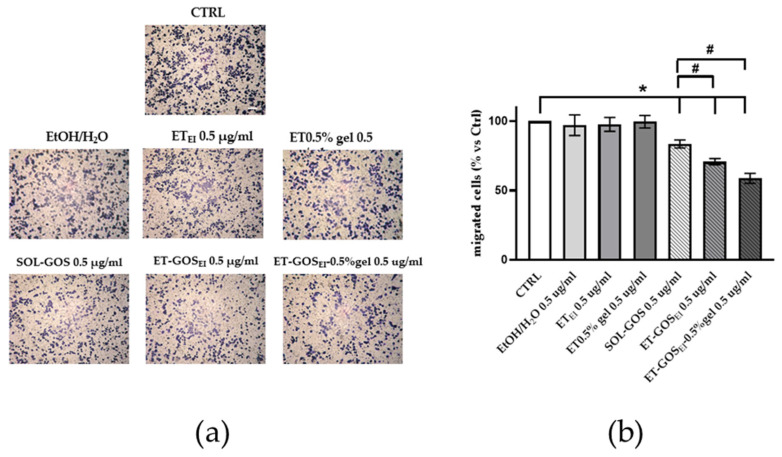
A375 melanoma cell migration after treatment with 0.5 µg/mL of GOS-loaded or unloaded formulations. (**a**) Representative images of A375 transwell migration after 24 h incubation with different formulations (scale bar 200 μm). (**b**) ImageJ quantification of migrated cells. Data are shown as average of 5 picture fields (20× magnification). * *p* < 0.05 treatment vs. CTRL; # *p* < 0.05 GOS-loaded vs. SOL-GOS by two-way ANOVA.

Since the in vitro scratch assay could not distinguish the effects of proliferation from migration, a transwell migration assay was conducted to better understand the possible role of GOS-loaded or unloaded formulations on melanoma cell migration. As shown in Figure 8, after 24 h of pretreatment of A375 melanoma cells with vehicle or with GOS-unloaded formulations, no migratory effect was observed, excluding any potential migratory effect due to the control formulations. Conversely, upon SOL-GOS, ET-GOS_EI_, and ET-GOS_EI_-0.5% gel treatment, a significant reduction in cellular migration was noticed. This effect was particularly significant in the case of ET-GOS_EI_-0.5% gel; indeed, in this case, the migratory ability was not only reduced when compared to the control condition, but also when compared to SOL-GOS and ET-GOS_EI_, confirming the delayed wound closure results reported above (Figure 8).

The in vitro antimelanoma efficacy of GOS loaded in a nanostructured gel was previously evaluated by Rajkumar and colleagues [11]. Particularly, the authors performed an MTT study on A375 cells for only 24 h; conversely, in the present study a wound healing assay was further conducted, associated with a transwell migration assay, demonstrating the possible role of GOS-loaded or unloaded formulations on melanoma cell migration. In addition, the possibility of employing ETs in the treatment of melanoma was previously evaluated by Yu and colleagues [43]. The authors described a mitoxantrone-loaded ET gel as an effective non-invasive melanoma therapeutic approach, avoiding the side effects due to the systemic administration of anticancer drugs. Furthermore, our research group previously investigated the in vitro potential of quercetin-loaded ETs and transethosomes, based on PC and polysorbate 80, in the treatment of melanoma. Particularly, in vitro permeation studies revealed an enhancement of quercetin permeation in the case of transethosomes compared to ETs [32].

## 4. Conclusions

This study presents a comprehensive investigation into the development and characterization of ET gels for the transdermal delivery of GOS, aimed at the potential treatment of cutaneous melanoma. Through a systematic pre-formulative study, the EI method was identified as the optimal technique for ET preparation, producing vesicles with a smaller size and enhanced EC compared to the WI method. The resultant ET-GOS_EI_ vesicles demonstrated effective control over GOS release in vitro, while improving its permeability through membrane models.

Moreover, the incorporation of x-gum yielded a semi-solid ET gel suitable for cutaneous application. Notably, the antioxidant activity of GOS was preserved upon loading into ETs, while biological assays on A375 melanoma cells revealed a pronounced inhibitory effect on cell migration and wound closure, particularly with the ET-GOS_EI_-0.5% gel.

The results of this study underline the promise of ET-GOS_EI_-0.5% gel as a targeted approach for melanoma treatment and possibly other skin diseases involving oxidative and inflammatory stress. As a future perspective, since most therapeutic approaches impact both tumor cells and the normal function of healthy tissues, an investigation into the effect of ET-GOS_EI_ 0.5% gel on healthy melanocytes, which has not been previously explored, could provide valuable insights. This research would help determine the feasibility of employing this GOS-loaded delivery system as an adjunct therapy for human melanoma. Furthermore, we could consider the possibility to load GOS into transethosomes to further enhance skin permeation. However, to translate our findings into clinical applications, further ex vivo and in vivo studies are warranted. These should focus on validating the therapeutic efficacy, assessing long-term safety profiles, and optimizing the formulation for scalability and patient compliance. Particularly, a comprehensive preclinical evaluation, preceded by a thorough investigation of the intrinsic biological activity of ET-GOS_EI_-0.5% gel, is necessary before its clinical implementation. The integration of nanotechnological innovations, such as ET forms, holds significant potential to advance the field of topical drug delivery and address critical challenges in dermatological oncology.

## Figures and Tables

**Table 1:** Composition of ETs produced by WI or EI methods.

Formulation	PC ^1^ (%, *w*/*w*)	Ethanol (%, *w*/*w*)	Water (%, *w*/*w*)	GOS ^2^ (%, *w*/*w*)
ET_WI/_ET_EI_	0.9	29.1	70	-
ET-GOS_WI_/ET-GOS_EI_	0.9	28.8	70	0.03

^1^: soy phosphatidylcholine; ^2^: gossypin.

**Table 2:** Size distribution parameters and entrapment capacity of indicated forms.

Formulation	Time (Days)	Z-Average (nm) ± s.d.	Dispersity Index ± s.d.	EC ^1^ (%)
ET_WI_	1	213.73 ± 9.72	0.150 ± 0.01	-
	60	243.20 ± 0.00	0.260 ± 0.00	-
ET_EI_	1	146.78 ± 4.10	0.192 ± 0.02	-
	60	155.33 ± 6.14	0.198 ± 0.00	-
ET-GOS_WI_	1	197.13 ± 11.43	0.148 ± 0.03	89.9 ± 7.5
	60	202.67 ± 5.45	0.163 ± 0.06	-
ET-GOS_EI_	1	148.70 ± 7.75	0.166 ± 0.02	94.3 ± 4.5
	60	165.25 ± 4.74	0.193 ± 0.02	-

^1^ Entrapment capacity calculated following Equation (2); s.d.: standard deviation.

**Table 3:** IVRT parameters and kinetic data of indicated formulations.

Formulation	R ^1^ (µg/cm^2^/h)	A ^2^ (µg/cm^2^)	Zero Order Plot (R^2^)	First Order Plot (R^2^)	Higuchi Plot (R^2^)	Peppas Plot (n/R^2^)
ET-GOS_WI_	10.77 ± 2.57	146.15 ± 5.15	0.968	0.973	0.909	1.46/0.950
ET-GOS_EI_	9.27 ± 1.70	147.60 ± 9.12	0.970	0.975	0.896	1.66/0.936
SOL-GOS	43.27 ± 7.14	199.14 ± 29.69	0.972	0.989	0.924	1.93/0.729

^1^: Release rate; ^2^: amount of GOS released after 24 h (ET-GOS_WI_ and ET-GOS_WI_) and 5 h (SOL-GOS); GOS concentration was 0.3 mg/mL; data are means of 6 independent Franz cell experiments ± s.d.

**Table 4:** IVPT parameters of indicated formulations.

IVPT Parameters	ET-GOS_WI_	ET-GOS_EI_	SOL-GOS
Jss ^1^ (μg/cm^2^/h)	0.241 ± 0.192	0.387 ± 0.267	0.077 ± 0.020
Kp ^2^ (cm/h) × 10^3^	0.737 ± 0.547	1.115 ± 0.600	0.248 ± 0.063
Tlag ^3^ (h)	1.498 ± 0.858	2.846 ± 0.667	1.894 ± 1.554
D ^4^ (cm^2^ h^−1^) × 10^5^	13.377 ± 7.662	6.141 ± 1.646	5.59 ± 10.936
P ^5^ membrane/vehicle	0.164 ± 0.035	0.550 ± 0.145	0.098 ± 0.096
A ^6^ (µg/cm^2^)	38.306 ± 4.057	35.452 ± 12.640	0.260 ± 0.000

^1^: Steady-state flux per unit area; ^2^: permeability coefficient; ^3^: lag time; ^4^: diffusion coefficient; ^5^: partition coefficient; ^6^: cumulative amount of GOS diffused at 24 h; GOS concentration was always 0.3 mg/mL; data are means of 6 independent Franz cell experiments ± s.d.

**Table 5:** Spreadability test results of reported gels.

Formulation	x-Gum % (*w*/*w*)	Spreadability ^1^ (g cm/s)
ET_WI_ gel	0.5%	23.33 ± 1.52
	0.75%	18.67 ± 0.58
	1%	17.67 ± 0.58
ET_EI_ gel	0.5%	24.01 ± 3.6
	0.75%	20.12 ± 1.0
	1%	18.33 ± 1.0

^1^: calculated as reported in Equation (6); data are the means of three independent measurements ± s.d.

**Table 6:** Results of DPPH test.

Formulation	DPPH IC_50_ (µg/mL)
ET-GOS_EI_	6.77 ± 0.283 *
ET-GOS_EI_-0.5% gel	10.625 ± 0.163 *
SOL-GOS	7.435 ± 0.403
SOL-AA	1.20 ± 0.096

Each value is the mean of at least three different experiments (mean ± SEM). * *p* < 0.005.

## Data Availability

Data are available from the authors upon request.

## Supplementary Materials

The following supporting information can be downloaded at: https://www.mdpi.com/article/10.3390/antiox14020186/s1, Figure S1: Size stability evaluated over 60 days during which formulations were stored at room temperature and protected from light. (a) Z-Average and (b) dispersity index measured after 1, 15, 30, and 60 days from preparation; Figure S2: SAXS profile of ET_WI_ (A) and ET-GOS_WI_ (B) obtained through temperature scan between 15 °C and 60 °C both in heating and cooling; Figure S3: IVRT (a) and IVPT (b) results from ET GOS_EI_ (red circles), ET GOS_WI_ (blue circles), SOL GOS (black crosses) and ET-GOS_EI_-0.5% gel (yellow squares) as determined by Franz cells. Data are the mean of 6 independent experiments ± s.d.; Table S1: Zeta potential values of the indicated forms; Table S2: IVRT and IVPT parameters of ET-GOS_EI_-0.5% gel formulation.
