# Supplementary material for: Gossypin-Loaded Ethosome Gel for Cutaneous Administration: A Preliminary Study on Melanoma Cells
Source: Antioxidants (Basel). 2025 Feb 5;14(2):186. doi: 10.3390/antiox14020186 (PMC11852004; doi:10.3390/antiox14020186)
Supplement: Supplementary file 1 [file antioxidants-14-00186-s001.zip › antioxidants-3429018-supplementary.pdf]

Supplementary Materials

Gossypin loaded ethosomal gel for cutaneous administration: a preliminary study on melanoma cells.

Agnese Bondi[1], Walter Pula[1], Mascia Benedusi[2], Giulia Trinchera[2], Anna Baldisserotto[3], Stefano Manfredini[3], Maria Grazia Ortore[4], Alessia Pepe[4], Paolo Mariani[4], Marc C. A. Stuart[5], Giuseppe Valacchi[6,7,8]* and Elisabetta Esposito[1]*

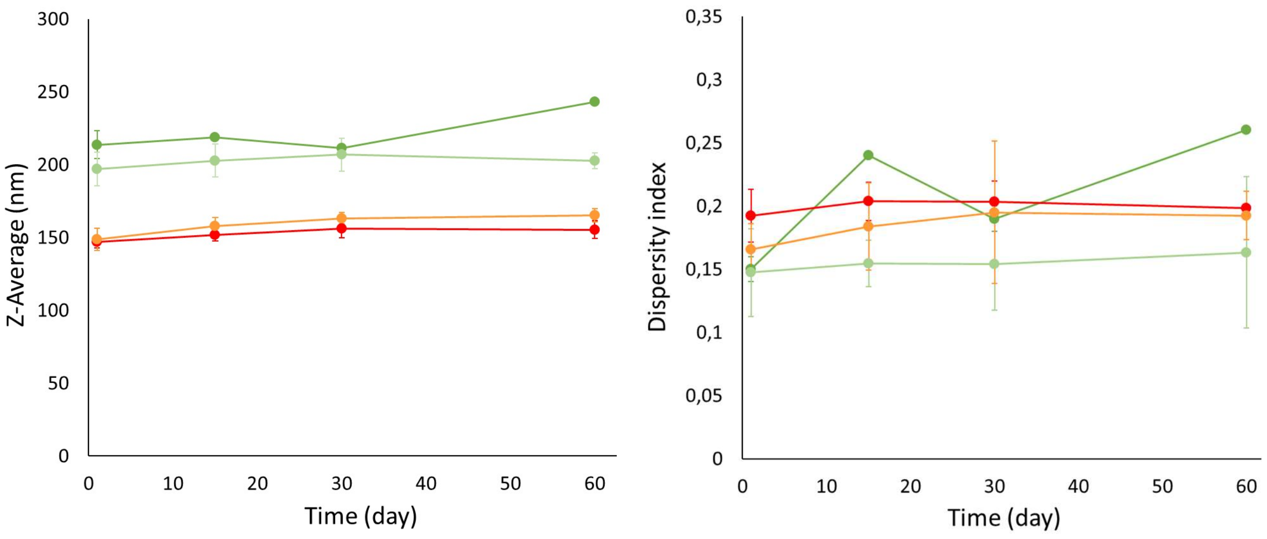

(**a**) (**b**)

Figure S1. Size stability evaluated over 60 days during which the formulations were stored at ambient temperature and protected from light. (a) shows Z-Average and (b) Dispersity index respectively measured after 1, 15, 30 and 60 days from preparation.

Small and Wide-angle X-ray scattering

The results obtained from the temperature scan are reported in Fig. S2, considering both sample ET$_{WI}$ (Fig. S2 A) and ET-GOS$_{WI}$ (Fig. S2 B). The result shown in Fig. S2 A regards only three curves to provide a clearer understanding and here two situations can be emphasized. On one side, the characteristical Bragg-peak has a lower intensity by increasing temperature, indicating that a possible alteration of some lipid lamellar of these vesicular nanosystems occurs, without destroying the ET structure. This aspect is also confirmed by the reversible nature of ET$_{WI}$ sample. Indeed, when the temperature returns to 15 °C by cooling down the sample, the peak shows the same starting position. Conversely, in the case of ET-GOS$_{WI}$ the SAXS profiles as a function of temperature are not altered, indicating that this formulation remains stable also at high temperature (see Fig. S2 B).

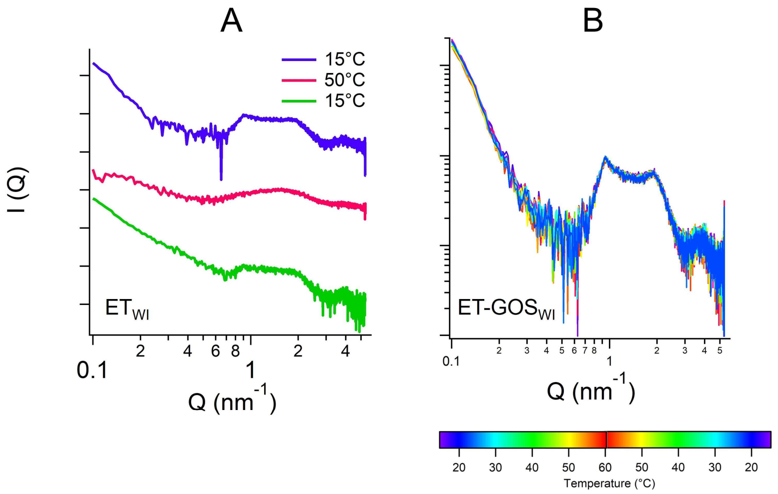

Figure S2. SAXS profile of ET$_{WI}$ (A) and ET-GOS$_{WI}$ (B) obtained through a temperature scan between 15 °C and 60 °C both in heating and cooling.

Table S1. Zeta potential values of the indicated forms.

Formulation	Z-Potential (mv)
ET$_{WI}$	-23.39 ± 0.21
ET$_{EI}$	-20.12 ± 2.54
ET-GOS$_{WI}$	-24.99 ± 2.21
ET-GOS$_{EI}$	-21.79 ± 7.60

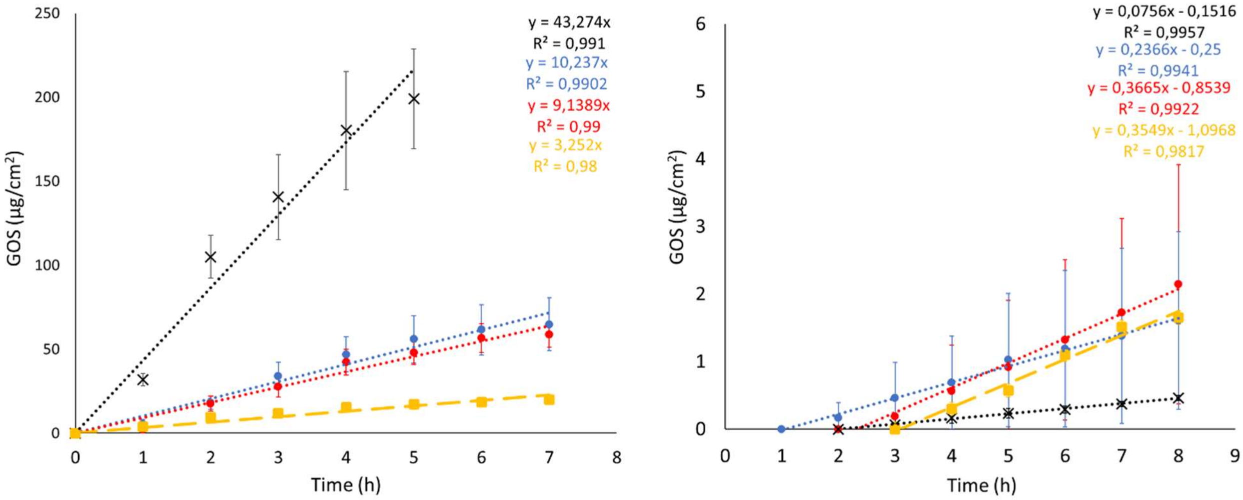

(a) (b)

Figure S3. IVRT (a) and IVPT (b) results from ET GOS$_{EI}$ (red circles), ET GOS$_{WI}$ (blue circles), SOL GOS (black crosses) and ET-GOS$_{EI}$-0.5%gel (yellow squares) as determined by Franz cells. Data are the mean of 6 independent experiments ± s.d.

Table S2. IVRT and IVPT parameters of ET-GOS$_{EI}$-0.5%gel formulation.

Parameters	ET-GOS$_{EI}$-0.5%gel
R[1] (μg/cm^2/h)	3.740 ± 0.590
Jss[2] (μg/cm^2/h)	0.355 ± 0.121
Kp[3] (cm/h) x 10^3	1.162 ± 0.242
Tlag[4] (h)	3.090 ± 0.525
D[5] (cm^2 h^{-1}) x 10^5	5.419 ± 2.242
P[6] membrane/vehicle	0.680 ± 0.123

1: Release rate: 2: Steady-state flux per unit area ; 3: permeability coefficient ; 4: lag-time ; 5: diffusion coefficient; 6: partition coefficient ; GOS concentration was always 0.3 mg/mL; data are the mean of 6 independent Franz cell experiments ± s.d.
